# Grading facial expression is a sensitive means to detect grimace differences in orofacial pain in a rat model

**DOI:** 10.1038/s41598-018-32297-2

**Published:** 2018-09-17

**Authors:** Megan M. Sperry, Ya-Hsin Yu, Rachel L. Welch, Eric J. Granquist, Beth A. Winkelstein

**Affiliations:** 1 Department of Bioengineering, University of Pennsylvania, Pennsylvania, USA; 2 Department of Endodontics, School of Dental Medicine, University of Pennsylvania, Pennsylvania, USA; 3 Oral & Maxillofacial Surgery, School of Medicine, University of Pennsylvania, Pennsylvania, USA; 4 Department of Neurosurgery, School of Medicine, University of Pennsylvania, Pennsylvania, USA

## Abstract

Although pre-clinical models of pain are useful for defining relationships between biological mechanisms and pain, common methods testing peripheral sensitivity do not translate to the human pain experience. Facial grimace scales evaluate affective pain levels in rodent models by capturing and scoring spontaneous facial expression. But, the Rat Grimace Scale (RGS) has not assessed the common disorder of temporomandibular joint (TMJ) pain. A rat model of TMJ pain induced by jaw loading (1 hr/day for 7 days) was used to investigate the time course of RGS scores and compare them between different loading magnitudes with distinct peripheral sensitivity profiles (0N–no sensitivity, 2N–acute sensitivity, 3.5N–persistent sensitivity). In the 3.5N group, RGS is elevated over baseline during the loading period and one day after loading and is correlated with peripheral sensitivity (ρ = −0.48, p = 0.002). However, RGS is not elevated later when that group exhibits peripheral sensitivity and moderate TMJ condylar cartilage degeneration. Acutely, RGS is elevated in the 3.5N loading group over the other loading groups (p < 0.001). These findings suggest that RGS is an effective tool for detecting spontaneous TMJ pain and that spontaneous pain is detectable in rats that develop persistent TMJ sensitivity, but not in rats with acute resolving sensitivity.

## Introduction

Pre-clinical rodent studies commonly assess pain by measuring hypersensitivity in the relevant dermatome for the pathology or etiology being studied^1^. However, that reflex outcome does not translate to the human experience of pain because it fails to capture the cortical and affective components involved in the perception of pain^1–4^. In many studies, peripheral sensitivity is measured using mechanical stimuli to illicit a withdrawal response, defining a threshold for sensitivity^5–10^. Although this method is useful for investigating some forms of sensitivity, it assesses only the evoked component of pain^3^ and nociceptive pain pathways^11^. Hypersensitivity measurements do not evaluate the presence or extent of the non-nociceptive pathways that influence the *experience* of pain, including corticolimbic interactions that produce negative affect, drive emotional learning, and reorganize circuitry^12^. Further, the use of mechanical and/or thermal hypersensitivity does not readily translate into clinical outcomes because hypersensitivity is identified *only* in a subset of patients who report pain^3^. In contrast, spontaneous pain is identified almost universally among chronic pain patients^3,13,14^ and has been identified as the principal symptom of clinical pain^13,15^. However, methods have recently been developed to measure spontaneous pain in pre-clinical animal models of inflammatory and neuropathic pain to improve translatability to the human experience^16–20^.

**Figure 1:**
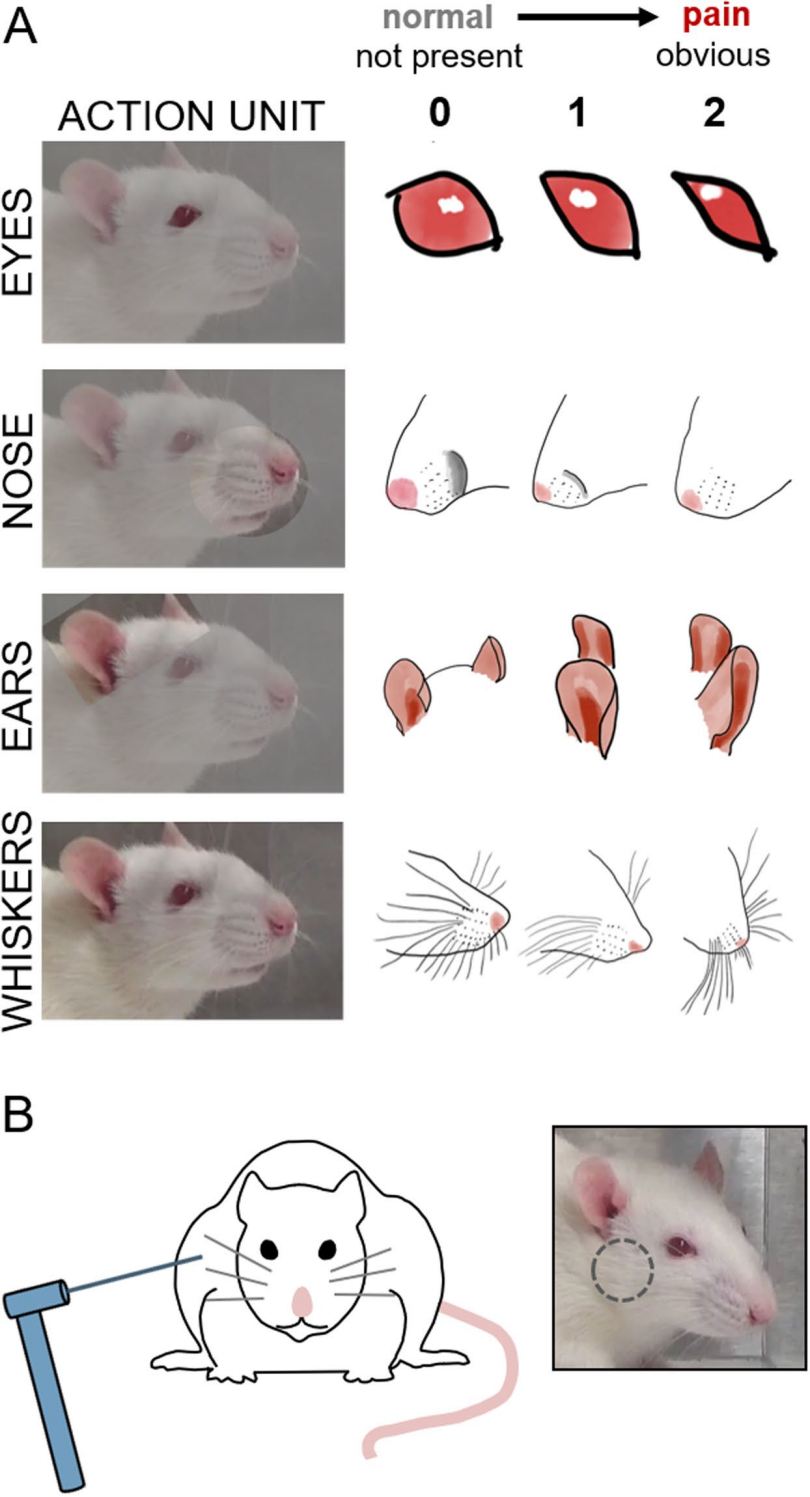
Sensitivity was measured by (**A**) RGS scoring of facial features, including the eyes, nose, ears, and whiskers, and (**B**) mechanical reflex testing in the TMJ region (designated by the grey circle).

In most patients, spontaneous pain is reported using pain scales^21,22^, questionnaires^23,24^, and descriptions of sensations^15,25^. However, those methods cannot be used for non-verbal populations; instead, facial expression has been adopted as a surrogate measure of spontaneous pain. The Facial Action Coding System transferred human facial expression movement into action units^26^ and the Neonatal Facial Coding System is now widely used in infant populations^27^. Pre-clinical pain researchers used these approaches as models to develop objective methods to evaluate if pain is present in animals^16^; the mouse grimace scale (MGS) adapted that scoring to utilize five facial features as indicators to evaluate pain^16^ and a four-feature facial coding system was also developed for rats (Fig. 1)^28^. The MGS and Rat Grimace Scale (RGS) both capture spontaneous pain and have been hypothesized to represent a measure of the animal’s affective response to pain^16,28^. Although the RGS has been used to assess pain in models of intraplantar complete Freund’s adjuvant (CFA) injection, intra-articular kaolin/carrageenan injection, plantar incision, laparotomy, experimental tooth movement, and acute chemotherapy-induced mucositis^11,17–19,28,29^, its use in orofacial pain has been limited to orthodontic tooth movement^17^. There are no studies investigating spontaneous pain of the temporomandibular joint (TMJ) using RGS as an assessment approach.

Orofacial pain near the jaw and ear can originate from mechanical dysfunction and/or low-grade inflammation of the TMJ, which articulates the mandible to the bones of the skull^30^. TMJ pain affects 5–12% of the population^31^ and can be a debilitating, chronic condition associated with both increased nociceptive activity and alterations in brain circuitry^30,32,33^. To date, studies have primarily relied on evoked measurements, such as mechanical hyperalgesia, to assess TMJ pain and the effect of potential therapeutics on TMJ sensitivity^5,7,34,35^. Based on the anatomic location of the TMJ, RGS is expected to be a particularly robust measure for pain in this region and may also be a useful tool for studying other orofacial pain syndromes. Surrogate measurements of spontaneous pain have been used in studies of orofacial pain, such as bite force, grooming behavior, guarding, and weight loss^1,11,35^. However, those behaviors are difficult to quantify, and present challenges in differentiating whether they indicate stress, pain, paresthesia, and/or avoidance behavior^36^.

**Figure 2:**
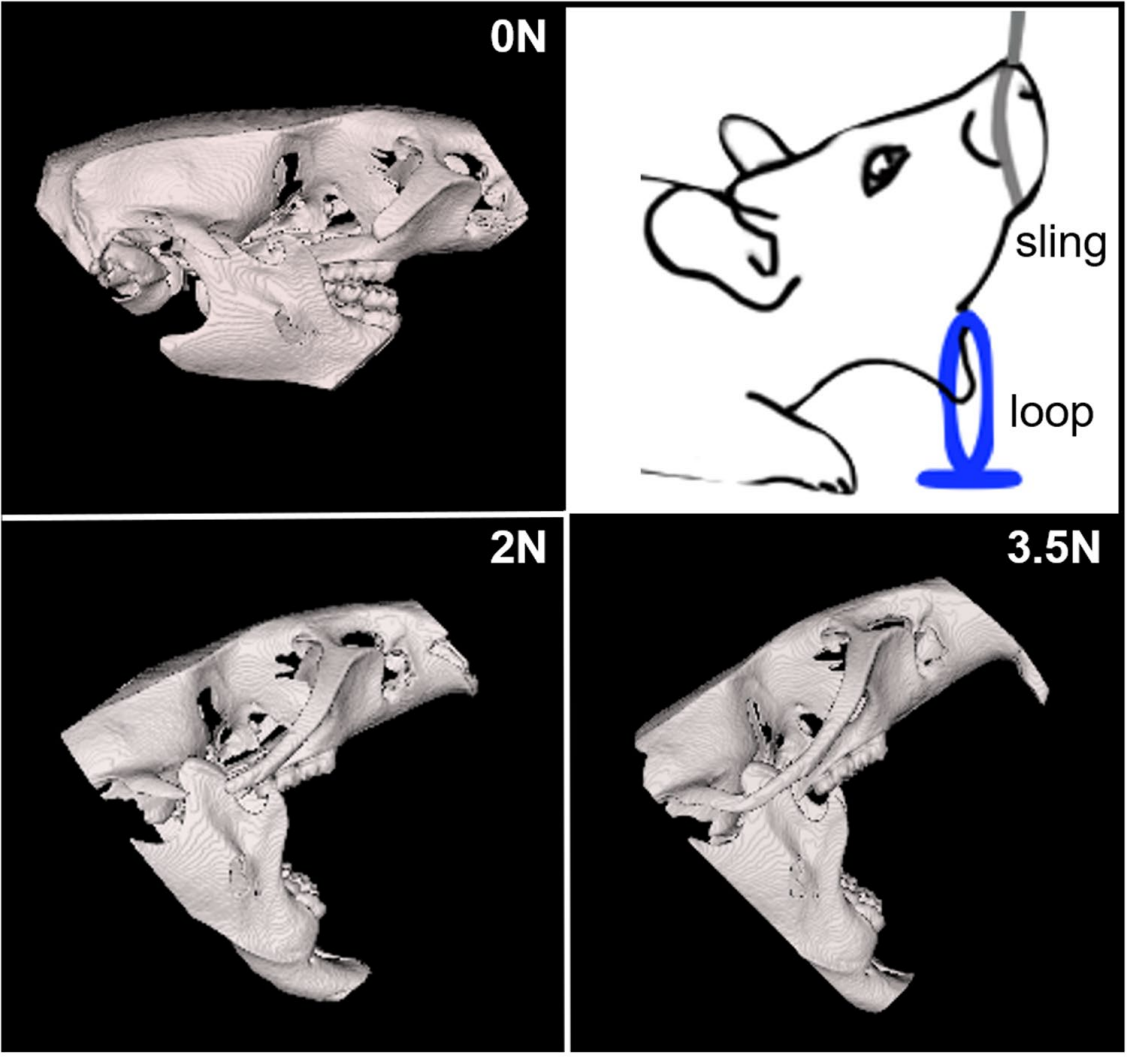
Three-dimensional CT reconstructions of the rat skull during the corresponding magnitudes of mechanical loading (0N, 2N, 3.5N) to the TMJ. The mandible was fixed by a nylon loop and the maxilla was held open by a sling with the applied load.

In this study, RGS was implemented to assess its ability to detect TMJ pain induced using repeated TMJ loading that produces moderate osteoarthritic pathology in the joint^5,7^. Assessments were measured at baseline before any procedures, during the days of the TMJ loading period, and after the loading period, and were compared with conventional mechanical reflex testing with von Frey filaments. RGS scores were also compared across separate groups of rats receiving different degrees of TMJ loading (0N, 2N, 3.5N) (Fig. 2), known to induce different reflex responses. Exposure to repeated TMJ loading at different magnitudes produces distinct TMJ sensitivity profiles evoked in response to von Frey filament stimuli^5,7^. Both the 3.5N and 2N groups are sensitive during and immediately after TMJ loading; however, after termination of loading, sensitivity returns to baseline levels in the 2N group whereas the 3.5N group remains sensitive for 8 days^5,7^. The 0N control group does not exhibit any TMJ sensitivity at any time. Although orofacial sensitivity is commonly assessed using evoked measurements, the relationship between evoked and spontaneous pain is untested in TMJ pain. In addition, TMJs were harvested at early and late time points after the cessation of loading to evaluate the extent, if any, of structural changes in the cartilage of the condyle using Safranin O/Fast Green staining and Mankin scoring. Despite the widespread use of Mankin scoring to evaluate cartilage degeneration in animal models of TMJ osteoarthritis^37–39^, the temporal relationship between TMJ pain and structural changes of the joint remains poorly understood.

## Results

### RGS scores increase for a painful insult but are only correlated to reflex responses acutely

**Figure 3:**
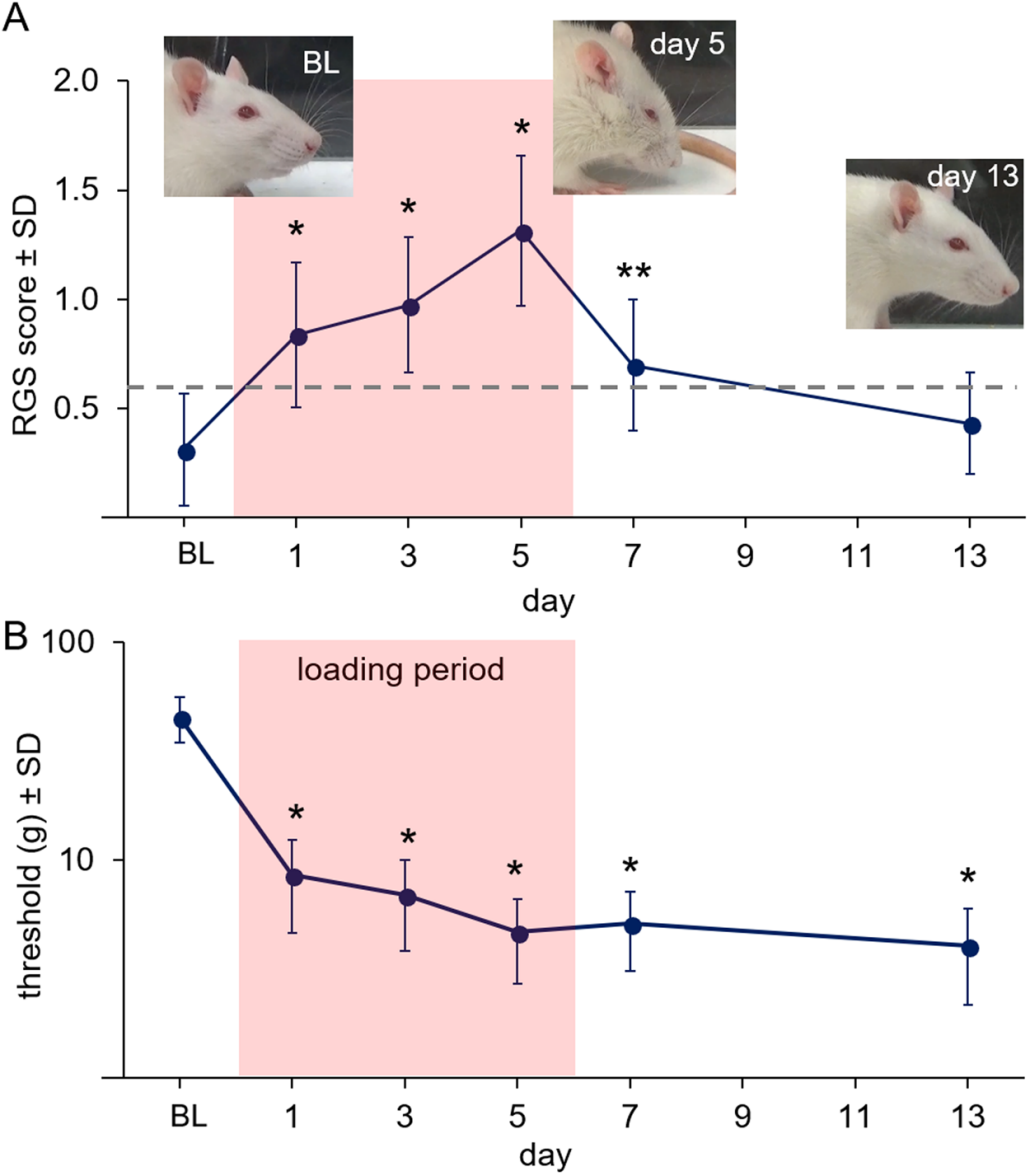
(**A**) RGS scores increase from baseline (BL) after 3.5N loading on days 1, 3, and 5 (*p < 0.001) and 1 day after the cessation of loading (day 7) (**p = 0.019). However, RGS scores return to baseline levels by day 13 (p = 0.820). The grey line indicates the previously reported analgesic intervention score for the RGS^46^. (**B**) Head withdrawal thresholds (g) decrease from BL levels both during and after loading on all days (*p < 0.01). The loading period is labeled in (**B**) and shown in the pink shaded region of both panels.

The RGS score was assessed in a rat model of TMJ loading that has been previously shown to induce persistent orofacial sensitivity as measured by mechanical reflex testing^5,7^. The RGS values reflect an overall rating based on presentation of four action units of the face (Fig. 1). In the rats undergoing 7 days of 3.5N loading (Fig. 2), the RGS scores 3 hours after exposure were elevated (p < 0.001) immediately from baseline (0.31 ± 0.25) on day 1 (0.84 ± 0.33), day 3 (0.97 ± 0.31), and day 5 (1.31 ± 0.35) (Fig. 3A). On day 7, which was 1 day after the last day of loading, RGS scores remained elevated (p = 0.019) over baseline levels (0.70 ± 0.30). However, at day 13, RGS scores returned to baseline levels (0.43 ± 0.23) and were not significantly different (p = 0.82) from those responses.

In contrast, head withdrawal thresholds were significantly different from baseline on all days they were assessed (p < 0.001). Thresholds were lower (p < 0.01) than baseline values (45 ± 11 g) during the loading period on day 1 (9 ± 4 g), day 3 (7 ± 3 g), and day 5 (5 ± 2 g), and remained significantly decreased from baseline withdrawal thresholds (p < 0.01) at day 7 (5 ± 2 g) and day 13 (4 ± 2 g) (Fig. 3B). Head withdrawal thresholds and RGS scores were not correlated (ρ = −0.28, p = 0.09) when all time points were included. However, withdrawal threshold and RGS were moderately and significantly correlated (ρ = −0.48, p = 0.002) when including times during loading (days 1, 3, and 5) and at the early time after its cessation (day 7).

### RGS scoring acutely detects differences that are not differentiated by reflex responses until later

**Figure 4:**
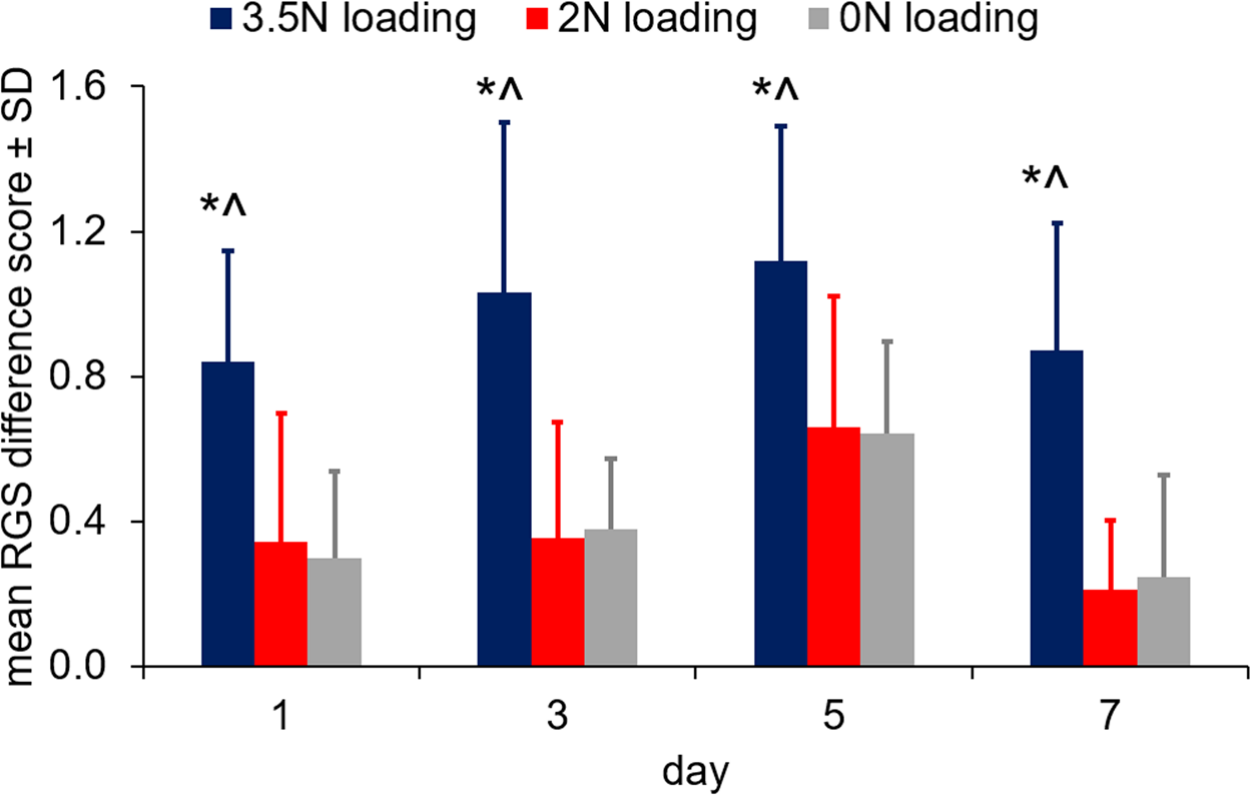
RGS scores are greater in the 3.5N loading group than either the 2N (*p < 0.05) or 0N (^p < 0.03) group during the loading period (days 1, 3, 5) and immediately after the cessation of loading (day 7). The scores for the 2N and 0N loading groups are not different from each other at any day.

We then measured RGS scores using different TMJ loading severities (3.5N, 2N, and 0N mouth-loading; Fig. 2) that have been shown to exhibit different TMJ sensitivity profiles after the cessation of loading^5,7^. Overall, RGS scores from the 3.5N loading group were significantly higher (p < 0.001) than each of the 0N and 2N groups across all days tested (Fig. 4). The RGS scores for the 0N and 2N groups were not different from each other (p = 0.999) on any day and on average increased 0.39 ± 0.32 from baseline on each of the test days. Peak differences in RGS from baseline were observed on day 5 in each of the groups: 3.5N (1.12 ± 0.37), 2N (0.66 ± 0.36), and 0N (0.64 ± 0.25) (Fig. 4). However, RGS scores at that time were not significantly different from the scores recorded at days 1 and 3. The most robust differences between groups were observed at day 7, with RGS difference scores for the 3.5N loading group (0.87 ± 0.35) elevated over those for each of the 2N (0.21 ± 0.19; p = 0.0003) and 0N (0.25 ± 0.28, p = 0.006) groups. RGS scores were significantly decreased from day 5 to day 7 only in the 2N group (p = 0.049).

### TMJ cartilage is altered after loading that acutely elevates RGS scores

**Figure 5:**
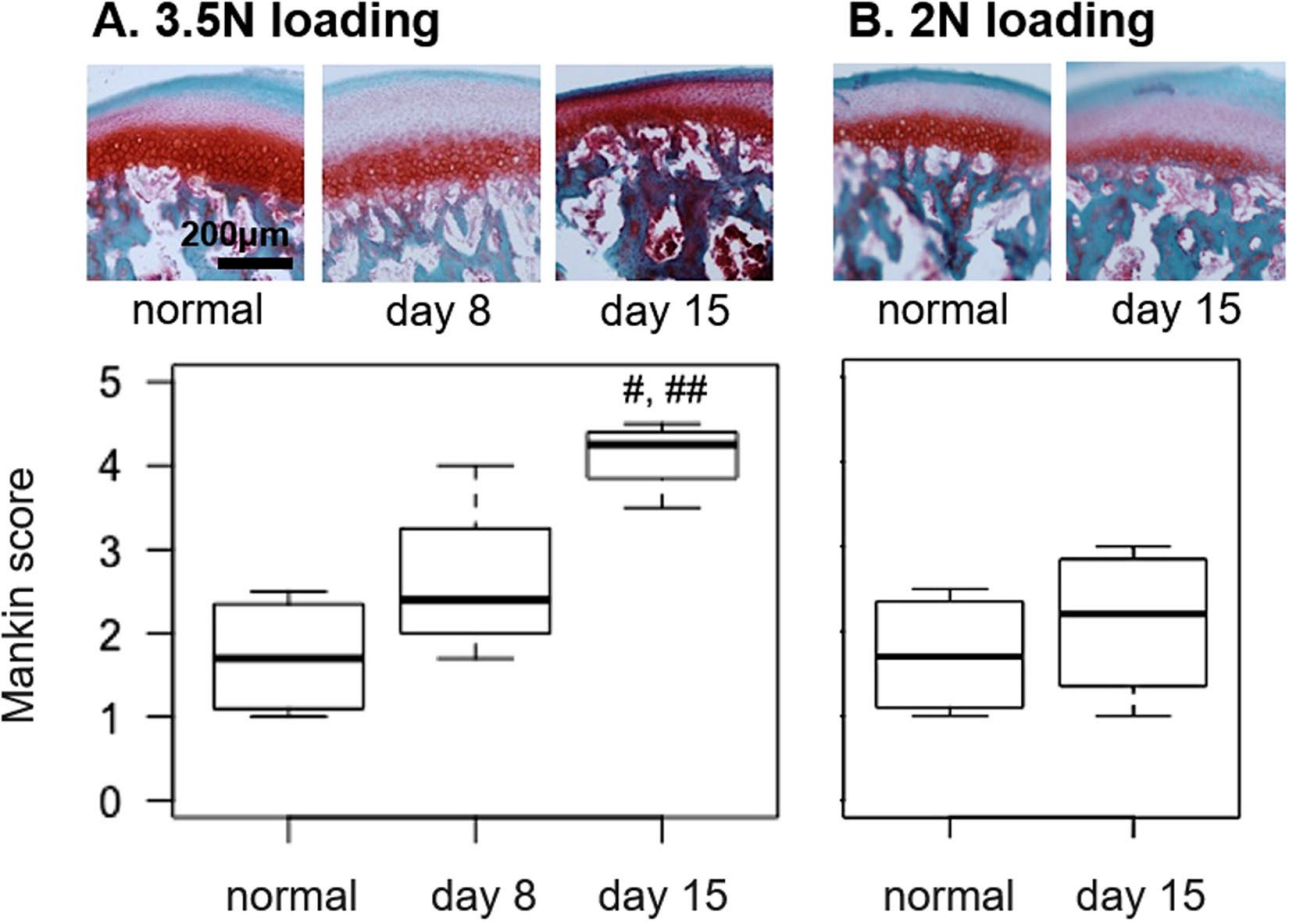
(**A**) Mankin scores of the TMJ condyle are unchanged at day 8 after 3.5N loading but increase at day 15 over normal (^#^p = 0.004) and day 8 levels (^##^p = 0.047). (**B**) Mankin scores are unchanged from normal levels at day 15 after 2N loading.

TMJ condyle cartilage was evaluated using Safranin O/Fast Green staining and the Mankin scoring system to determine if RGS scores parallel changes in joint structure that are associated with cartilage osteoarthritis^37,40^. Mankin scores for the 3.5N loaded TMJs were greater at day 15 (4.10 ± 0.37) than scores for normal tissue (1.71 ± 0.64; p = 0.004) and those at day 8 (2.60 ± 0.86; p = 0.047) after 3.5N loading (Fig. 5). Mankin scores for the 2N loaded TMJs at day 15 (2.1 ± 0.92) were not different (p = 0.548) from the scores for normal TMJs (Fig. 5), indicating no structural changes in that model.

## Discussion

This study used the RGS to assess spontaneous pain in a rat model of TMJ pain and compared RGS between groups with different TMJ sensitivity profiles to evaluate its ability to differentiate pain states (Figs 3 and 4). Although facial grimace scales have been used to characterize visceral, inflammatory, and neuropathic pain in rodent models of skin and joint inflammation, cervical radiculopathy, peripheral neuropathy, bladder infection, and abdominal constriction^16,18,19,41^, this is the first report of facial grimace for TMJ pain and in the context of repeated joint over-loading, which is a major cause of joint degeneration and pain^42–45^. Since RGS is elevated over baseline during the TMJ loading period and one day after its cessation (Fig. 3), affective components of pain appear to be detectable both acutely after a single joint loading and also throughout repeated loading sessions. These same time points also exhibit sensitivity detected by reflex testing (Fig. 3), indicating that both nociceptive and spontaneous pain are present within this acute time frame. RGS scores during loading are elevated over the analgesic intervention score (RGS = 0.67), which has been previously determined as the quantitative standard separating painful from non-painful states^46^. However, unlike reflex testing which detects TMJ sensitivity one week after cessation of loading (day 13)^7^, RGS is *not* elevated (Fig. 3). This difference between outcomes suggests that RGS may be sensitive enough to serve as an early detector of persistent TMJ pain and may even be predictive of long-term differences in TMJ sensitivity (Fig. 3).

The difference between RGS scores and reflex thresholds at day 13 is consistent with the finding that RGS scores return to baseline within 48 hours of neuropathic injury^19^ and acute inflammation^18^. Certain inflammatory conditions, like those simulated by a CFA injection into the masseter muscle, elevate RGS scores for up to 3 days after that initiating event^41^. Although RGS most sensitively detects pain at acute time points^16,18,19,28^, the disappearance of facial grimace is not necessarily indicative of the resolution of spontaneous pain^28^. Natural adaptations have led prey species, like rodents, to inhibit facial grimace as soon as possible so that they do not become the target of predators^47^. By hiding painful expressions, spontaneous TMJ pain may persist for longer than is detectable by RGS. In fact, this hypothesis has been suggested previously for visceral, inflammatory, and neuropathic conditions^19,28^. Other tests of spontaneous behaviors, like conditioned place preference and activity monitoring, also fail to capture robust pain behaviors after acute time points^48^. Given these limitations, long-term monitoring of spontaneous pain remains challenging for chronic conditions.

Although the sensitivity of RGS is limited to acute times^16,19,28^, RGS is differentially modulated in the current study between groups, and is only elevated in the group that exhibits persistent TMJ sensitivity (3.5N loading) after the insult is removed (Fig. 4). The fact that this response is only observed in the group with persistent pain^5,7^ suggests a relationship between early facial grimace and long-lasting TMJ sensitivity. Inflammatory and incisional pain models demonstrate similar early facial grimace and hypersensitivity^18^; however, little attention has been paid to the connection between heightened RGS and long-lasting peripheral sensitivity^19^. Conversely, for the 2N loading group in which TMJ sensitivity resolves after the loading period^5,7^, facial grimace is not different from responses in the group receiving only anesthesia (0N loading) and exhibiting no TMJ sensitivity at any time point^5,7^. Together, the differential TMJ pain models used here represent a range of pain states: combined peripheral nociception and affective pain (3.5N loading), peripheral nociception that resolves (2N loading), and no pain (0N loading). The combined use of facial grimace and peripheral reflex testing in these models, as well as in incisional, inflammatory, and neuropathic pain models, emphasizes that when spontaneous pain is detectable, hypersensitivity is also present; however, hypersensitivity can be present in the absence of measurable spontaneous pain^18,19^. Although the RGS scores for the groups without reflex responses (2N and 0N) are not different (Fig. 4), this study only evaluated RGS scores at two time points after a stimulus: 3 hours and 24 hours (on day 7) after loading and anesthesia. It is possible that these selected time points may not capture the peak differences between groups, due to either anesthesia effects^19^ or a delayed onset of peak spontaneous pain^16^. Since differences between injured and anesthesia-only groups have been reported to peak within 3 to 9 hours after anesthesia^18,19,28^, more pronounced differences between such groups may be evident at later times when anesthesia effects have further subsided.

In addition to evaluating limited acute time points, this study also restricted evaluation to a single long-term time point (day 13). Because TMJ structure is *slowly* altered due to repeated jaw loading, with moderate thinning and cellular disorganization of the TMJ cartilage apparent by day 15 (Fig. 5), it is possible that RGS scores may increase again at later times when cartilage damage worsens. In fact, destabilization of knee ligaments in rats, which leads to synovial inflammation and cartilage destruction, has been shown to increase RGS scores 14 days after the initial surgery^49^, suggesting that spontaneous pain in osteoarthritis conditions may have a slower onset. Detection of delayed and episodic pain is particularly relevant to patients with painful TMJ disorders, who often experience cycles of pain ranging from a dull, constant ache to severe pain brought on by movement of the mandible^30^. As such, measuring both evoked and spontaneous pain over longer periods may provide clinically relevant information for accurately modeling both the nociceptive and affective components of TMJ pain and enable testing new treatments through these episodes.

This study is also limited by assessing only a *single* sex and strain of rat. TMJ pain was investigated in female rats because patients who seek care for TMJ pain are disproportionately female, with a female-to-male ratio between 3:1 and 9:1^30^. However, no sex differences have been identified using RGS measurements collected after intraplantar inflammatory injection or laparotomy^28^, suggesting that facial grimace may not be inherently different between sexes. It is possible that TMJ loading and/or pain presents with different effects in male and female rats since there are known sex differences in the mechanisms of immune-neuronal interaction^50^. Therefore, measuring RGS in male rats using this model would be helpful in determining if the associated affective pain responses are consistent across sexes.

Although osteoarthritic changes in the TMJ have been associated with increased orofacial sensitivity and reduced bite force^35,51^, this is the first study to investigate the relationship between joint structure and affective pain. Degeneration of the TMJ cartilage is only detectable in those rats with acute spontaneous pain and persistent evoked sensitivity (Figs 3–5), which suggests that early affective pain may be associated with, or even predict, structural changes in the joint that are associated with its overloading. However, the fact that structural changes develop *after* spontaneous pain appears to resolve suggests that early spontaneous pain may be driven by factors other than the structural modifications. In fact, the acute pain may be due to the increased intraarticular inflammatory factors that have been reported in the 3.5N loading group^7^. Direct induction of intraarticular inflammation, as by TNFα or CFA injection in the TMJ, has also been reported to acutely increase neuronal activity in the trigeminal subnucleus caudalis and to induce joint tissue degeneration^52,53^. However, inhibiting inflammatory factors in the TMJ is needed to further test the association between affective pain and acute TMJ inflammation after repeated joint loading.

Overall, this study not only supports the use of, but highlights the need for using, the RGS to monitor spontaneous pain in mechanical joint loading models of TMJ pain. Further, heightened RGS scores after TMJ loading appear to portend persistent TMJ peripheral sensitivity. As such, RGS may be a helpful modality for forecasting extended periods of peripheral sensitivity and for identifying clinical pain states, in TMJ and possibly other models of clinical pain. Although the non-invasive, unprovoked collection of RGS data is simple to execute, its scoring can be a labor-intensive process^28^. Recent advances in real-time RGS^20^ and convolutional neural networks for automated facial grimace scoring^54^ provide methods to streamline its use in both clinical and pre-clinical research applications. Therefore, more detailed investigation of the relationships between early facial grimace and persistent peripheral sensitivity would be valuable for real-time decision making in many environments. Despite strong evidence of on-going evoked sensitivity after the resolution of facial grimace across multiple models of clinical pain^18,19,41^, it is not yet known if acutely heightened RGS is predictive of persistently evoked sensitivity for other types of pain, particularly in models with tunable reflex responses. Although additional studies are needed to investigate the predictive value of acute RGS more broadly, this study demonstrates that spontaneous pain is sensitive enough for early and reliable detection of conditions that will develop persistent TMJ sensitivity, but not those with acute resolving sensitivity.

## Methods

### Animals

All studies used adult female Holtzman rats (HsdHot:Holtzman Sprague Dawley), weighing 268 ± 21 g at the start of the study (obtained from Envigo (Indianapolis, IN)). Rats were housed in groups of 2–3 in standard polycarbonate caging (AnCare; Bellmore, NY), with 0.25-inch corncob bedding (Bed-o’Cobs; The Andersons Lab Bedding Products; Maumee, OH) and ad libitum access to food (LabDiet 5001; LabDiet; St Louis, MO) and water (acidified to pH = 3). Rats were housed in an Association for Assessment and Accreditation of Laboratory Animal Care accredited vivarium under a 12:12 hour light:dark cycle in a temperature-controlled environment in accordance with recommendations set forth in The Guide for Care and Use of Laboratory Animals (8th edition)^55^. All procedures were approved by the University of Pennsylvania Institutional Animal Care and Use Committee and adhered to the guidelines for research and ethical issues of the International Association for the Study of Pain^56^.

The RGS scores were first measured in rats (n = 8) undergoing the TMJ loading that is known to induce sustained sensitivity in the TMJ region as assessed by mechanical reflex testing^7^. Facial grimace was assessed in those rats undergoing daily mouth-opening by 3.5N to determine if and when it is present after TMJ loading, as well as to directly compare RGS score with the time course of peripheral sensitivity. Based on those findings, the RGS was then compared between groups (n = 8/group) with different TMJ loading severities (0N, 2N, and 3.5N), which are known to have different peripheral sensitivity profiles^5,7^.

### Rat Grimace Scale evaluation & scoring

Facial grimace temporal patterns were evaluated at baseline (before procedures) and then daily using digital video recordings and RGS scores. When measurement corresponded to days of loading or other procedures requiring anesthesia, digital video recordings were acquired at 3 hours after exposure to isoflurane in order to allow the rats recovery time after anesthesia. All recording and monitoring procedures were performed in a quiet environment, and personnel remained out of visual contact with the rats for the duration of each session. Rats were placed singly in a 23 × 10 × 10 cm^3^ transparent Plexiglas chamber with a removable stainless-steel top. A digital video camera (Sony HDR-CX380/B High Definition Handycam) was placed in front of the wider side of the box. Rats were videotaped for 30 minutes and videos were saved as mp4 files. In the subset of rats known to have sustained TMJ sensitivity (3.5N loading), digital videos were acquired at baseline, every other day during the loading period (days 1, 3, and 5), and after loading on days 7 and 13 to assess long-term responses. To compare between groups with different sensitivity profiles (3.5N, 2N, and 0N), digital videos were acquired at baseline, during loading (days 1, 3, and 5), and after loading on day 7.

Ten images were captured from each 30-minute video session at 3-minute intervals as portable network graphic (PNG) files. In order to be included in grading and analysis, each image was required to have a clear view of the four action units (eyes, nose/cheek, ears, and whiskers) (Fig. 1) and to not be taken during grooming, sleeping, or active sniffing activity^16,19,28^. In the event that an image did not meet the requirements for extraction at the 3-minute interval, the video was advanced to the next immediate time point when an image could be used. The image-capture operator was blinded to the group, time point and rat identification. All images were randomized prior to scoring.

The scorer was given training using the standard scoring method^19^, before scoring any of the images from this study. Each image was scored for the action units: orbital tightening, nose/cheek flattening, ear curling, and whisker bunching (Fig. 1)^19,28^. For each image, the scorer assigned an intensity value of 0 (absent), 1 (moderately appearing), or 2 (obviously present) for each of the four action units. If the action units of the image could not be scored by the rater, the value would be assigned as “not scored” and not included in the average value for that image. The RGS score for each image was taken as the average of the action unit scores. The mean RGS score at an individual time point for each rat was the average of the RGS scores across the 10-images acquired. All images included in this study were scored by a single observer who was blinded to the procedures.

### Mechanical reflex testing

Mechanical reflex testing was performed at baseline before any procedures and every other day during and after loading (days 1, 3, 5, and 7). In a subset of rats, reflex testing was also performed at day 13 to assess later effects at times after the loading protocol and acute TMJ sensitivity. Reflex testing was performed at 8:00 AM each morning, before any other procedures. Thresholds for eliciting a head-withdrawal was evaluated using a series of von Frey filaments with increasing strengths from 0.6 g to 60 g (Stoelting; Wood Dale, IL)^5,7^. Three rounds of reflex testing were performed on the bilateral TMJs, with a 10-minute rest period between each round; each von Frey filament was applied five times to each side. If three of the five stimulations elicited a head withdrawal or immediate pawing of the stimulated areas, a positive response was recorded. The lowest-strength filament evoking a response was taken as the sensitivity threshold only if the next higher filament also elicited a response. If a rat was unresponsive to all filaments, the maximum filament strength (60 g) was recorded as the threshold.

### Procedures for TMJ loading

TMJ sensitivity was induced by mechanical loading of the jaw by mouth-opening^5,7^. Briefly, rats were anesthetized with isoflurane inhalation anesthesia (4–5% for induction; 2–3% for maintenance) mixed with oxygen during loading. Rats were placed in a ventilated acrylic chamber in the prone position. The mandible was held stationary with a nylon loop and the maxilla was opened by a sling attached to 0N, 2N, or 3.5N load for 1 hour/day (Fig. 2), applied daily for 7 continuous days (days 0 to 6). Rats were monitored during recovery from anesthesia. Weight and activity were monitored daily; no decreases in weight (>10% body weight) or activity were observed during the course of the study.

### Safranin O/Fast Green staining & Mankin scoring of the TMJ

Cartilage structure was assessed in naïve TMJs, 2N-loaded TMJs at day 15, and 3.5N-loaded TMJs at days 8 and 15 (n = 4/group). Rats were anesthetized with sodium pentobarbital (65 mg/kg), perfused with phosphate buffer saline (PBS), and fixed by 4% paraformaldehyde perfusion. TMJs were harvested, stored in 30% sucrose in PBS at 4 °C, and later decalcified using 0.25 M ethylenediaminetetraacetic acid for 3 weeks at 4 °C. Samples were embedded in Tissue-Tek OCT Compound (Saukura Finetek; Torrance, CA), sagittally sectioned (18 µm thickness), and thaw-mounted onto slides (3 sections/slide) to assess TMJ structure. TMJs were stained with Safranin O/Fast Green (Sigma Aldrich; St. Louis, MO) to evaluate morphological changes in the articular cartilage early after loading (day 8) and at a later time point (day 15). Stained TMJs were imaged at 10X magnification using the EVOS FL Auto Imaging System (Thermo Fisher Scientific, Waltham, MA). Cartilage degradation was measured by two blinded observers using the modified Mankin scale^37,40^. The Mankin scale scores cellular and background staining, chondrocyte arrangement, and structural condition of the cartilage, with a score of 0 for normal cartilage and 10 for maximally degenerate cartilage^37,40^.

### Data analyses & statistics

All data are expressed as mean ± standard deviation. The mean and standard deviation of RGS scores were calculated across all rats in each group for each time point. A repeated measures ANOVA tested for differences between days in RGS measurements for the 3.5N group.

The average withdrawal threshold for each rat was calculated from the three thresholds recorded for the left and right TMJ regions (6 measurements total) at each time. The mean and standard deviation of reflex thresholds were calculated across all rats in the 3.5N loading group for each day. Due to a non-normal distribution of the mechanical reflex data for the 3.5N loading group, the Kruskal-Wallis rank sum test was used to compare head withdrawal threshold between days in that group and the threshold at a given day was specifically compared to baseline with the pairwise Wilcoxon rank sum test, corrected for multiple comparisons. For that same group, the Spearman correlation coefficient was calculated between RGS and head withdrawal thresholds to quantify the relationship between those two measurements.

When comparing RGS values between loading groups, an RGS difference score was determined by subtracting the baseline scores from the scores measured at a given time point. Difference scores were compared between groups using a repeated measures ANOVA with day and group as factors. A Tukey post-hoc test compared groups on specific days. All statistical tests were performed in R software (version 3.2.3, The R Foundation for Statistical Computing) and results were considered statistically significant at p < 0.05.

A one-way ANOVA compared Mankin scores for the 3.5N loading group across time points and relative to normal tissue. A t-test compared normal Mankin scores to the 2N loading group at day 15.

## Data Availability

The datasets generated during and/or analyzed during the current study are available from the corresponding author on reasonable request.
